# Inequalities in access to healthcare by local policy model among newly arrived refugees: evidence from population-based studies in two German states

**DOI:** 10.1186/s12939-021-01607-y

**Published:** 2022-01-24

**Authors:** Judith Wenner, Louise Biddle, Nora Gottlieb, Kayvan Bozorgmehr

**Affiliations:** 1 Present Address: Department of Population Medicine and Health Services Research, School of Public Health, Bielefeld University, PO Box 10 01 31, 33501 Bielefeld, Germany; 2 Section for Health Equity Studies and Migration, Department of General Practice and Health Services Research, University Hospital Heidelberg, Im Neuenheimer Feld 130.3, 69120 Heidelberg, Germany; 3 Department of Health Care Management (H80), Technical University Berlin, Strasse des 17. Juni 135, 10623 Berlin, Germany

**Keywords:** Access to healthcare, Health inequalities, Refugees, Asylum seekers, Healthcare utilization, Unmet needs, Emergency department use, Avoidable hospitalization, Germany

## Abstract

**Background:**

Access to healthcare is restricted for newly arriving asylum seekers and refugees (ASR) in many receiving countries, which may lead to inequalities in health. In Germany, regular access and full entitlement to healthcare (equivalent to statutory health insurance, SHI) is only granted after a waiting time of 18 months. During this time of restricted entitlements, local authorities implement different access models to regulate asylum seekers’ access to healthcare: the electronic health card (EHC) or the healthcare voucher (HV). This paper examines inequalities in access to healthcare by comparing healthcare utilization by ASR under the terms of different local models (i.e., regular access equivalent to SHI, EHC, and HV).

**Methods:**

We used data from three population-based, cross-sectional surveys among newly arrived ASR (N=863) and analyzed six outcome measures: specialist and general practitioner (GP) utilization, unmet needs for specialist and GP services, emergency department use and avoidable hospitalization. Using logistic regression, we calculated odds ratios (OR) and 95% confidence intervals for all outcome measures, while considering need by adjusting for socio-demographic characteristics and health-related covariates.

**Results:**

Compared to ASR with regular access, ASR under the HV model showed lower needs-adjusted odds of specialist utilization (OR=0.41 [0.24-0.66]) while ASR under the EHC model did not differ from ASR with regular access in any of the outcomes. The comparison between EHC and HV model showed higher odds for specialist utilization under the EHC model as compared to the HV model (OR=2.39 [1.03-5.52]). GP and emergency department utilization, unmet needs and avoidable hospitalization did not show significant differences in any of the fully adjusted models.

**Conclusion:**

ASR using the HV are disadvantaged in their access to healthcare compared to ASR having either an EHC or regular access. Given equal need, they use specialist services less. The identified inequalities constitute inequities in access to healthcare that could be reduced by policy change from HV to the EHC model during the initial 18 months waiting time, or by granting ASR regular healthcare access upon arrival. Potential patterns of differences in GP utilization, unmet needs, emergency department use and avoidable hospitalization between the models deserve further exploration in future studies.

**Supplementary Information:**

The online version contains supplementary material available at 10.1186/s12939-021-01607-y.

## Background

Providing access to healthcare for asylum seekers and refugees (ASR) is part of the receiving countries’ legal responsibilities [1]. However, many countries restrict the access of newly arriving ASR to regular healthcare services [2]. This may hinder need-based healthcare utilization, impact the health of ASR, and lead to inequalities in health [3, 4]. Monitoring access to healthcare of ASR is therefore an important public health task.

There are multiple ways of looking at access. Following Aday and Andersen [5], access to healthcare means that those “who need care get into the system” (p. 218). It can be measured as *potential access* [6], with a focus on availability of services and insurance coverage. However, this tells us little about the actual utilization and ignores the impact of social determinants beyond health system characteristics on healthcare utilization [7, 8]. Alternatively, access can be equated with *realized access* [6]. It can then be conceptualized as actual use of healthcare services (using utilization indicators) or as non-realized access (using outcome indicators like forgone care and unmet needs) [9]. Further measures include ambulatory care sensitive hospitalization, which measures the lack of timely provision of ambulatory treatment leading to a potentially avoidable hospitalization [10].

When looking at determinants of realized access to healthcare among newly arriving ASR, the legal framework must be considered. In Germany, newly arriving asylum seekers are excluded from statutory health insurance (SHI). Their health entitlements are regulated by the asylum seekers’ benefits act (in German: Asylbewerberleistungsgesetz (AsylbLG)), which is a federal law. Compared to SHI, the AsylbLG grants only a limited scope of healthcare during the first 18 months (15 months at time of data collection) or until a permanent protection status (refugee status or subsidiary protection) has been granted. The restricted entitlements include healthcare in case of acute illnesses and pain, preventive services and vaccines, and services related to pregnancy and birth (AsylbLG Art. 4). Access to further, mostly specialized, services can be granted on a case-by-case basis (AsylbLG Art. 6).

Given Germany’s federal structure and decentralized governance, the local governments and social welfare offices (SWO) are responsible for implementing the AsylbLG, and thus for organizing access to healthcare for ASR [11]. Different policy choices on state and local levels have led to two different access models being applied across Germany to implement the AsylbLG during the waiting period: the healthcare voucher (in the following: HV) and the electronic health card (in the following: EHC) [12, 13].

After the 18 months waiting period, asylum seekers under the AsylbLG are entitled to a scope of healthcare that is equivalent to SHI (AsylbLG Art. 2). Upon obtaining temporary or permanent legal status (before or after completion of 18 months), refugees have access to healthcare via SHI membership [11]. In both cases, they use a health insurance card to access health services. Hence, in practice, both recognized refugees and asylum seekers after 18 months in Germany have regular access, i.e. SHI- or SHI-equivalent entitlements to healthcare. For the purpose of our study, we thus distinguish between three different access models (c. Table 1):

1. **Healthcare vouchers** (**HVs**), which allow for healthcare access during the first 18 months in Germany. HVs are issued by the local SWO and entitlements are restricted (AsylbLG Art. 4 and 6). The paper-based HVs are usually valid for three months, or a single visit to a healthcare provider, and to be used within the respective administrative district. They are deposited with one service provider per calendar quarter, and each referral necessitates the approval and dispensation of another HV by the local SWO [11].
2. **Electronic health cards** (**EHCs**), which allow for healthcare access during the first 18 months in Germany. The EHCs are issued by a local statutory health insurance (SHI) fund, but financed by the SWOs. Entitlements are restricted (AsylbLG Art. 4 and 6). The EHC is issued once and then usually valid for the whole period of restricted entitlements. It has a digital record of patient details and stays with the patient. Though EHC holders do not become members of the SHI, the SHI carries out billing and accounting procedures against an administrative fee.
3. Irrespective of the access model used during the 18 months waiting period (HV or EHC), restrictions on healthcare entitlements and access are lifted after 18 months by granting SHI-equivalent health benefits (regulated by the AsylbLG Art. 2 and financed through the SWOs); or earlier if full SHI membership is granted through a temporary or permanent residence permit (**regular access**) [13–16].

**Table 1:** Overview of access policies for asylum seekers and refugees in Germany

Access Policies	Health care voucher	Eletronic health card upon arrival	Regular access	
Duration of stay	≤ 18 Months	≤ 18 Months	> 18 Months	≤ 18 Months
State of the asylum application	Ongoing	Ongoing	Ongoing or (temporary/permanent) residence permit granted	(Temporary/permanent) residence permit granted
Entitlement	Restricted (Art. 4 and 6 AsylbLG)	Restricted (Art. 4 and 6 AylbLG)	No restrictions, equivalent to social health insurance (Art. 2 AsylbLG)	
Federal State	Baden-Württemberg	Berlin	Baden-Württemberg and Berlin	

The choice of the access model during the first 18 months – HV oder EHC – has been subject to controversial political debates. Proponents of the EHC model claim that it reduces discrimination against ASR, facilitates need-based healthcare utilization and reduces the administrative workload for welfare offices and service providers. Proponents of the HV model caution that the EHC model will lead to excessive healthcare utilization and thereby increase expenses [13, 17, 18].

So far, there is limited evidence on the impact of the local policy model on access to healthcare. Qualitative studies suggest that the HVs are difficult to handle for healthcare users and providers and thereby hamper access to health services [18–21]. Quantitative studies provide further evidence for the disadvantages of the HV and of the entitlement restrictions during the 18-month waiting period; for instance, in terms of higher medical costs [22–25]. So far, there is no quantitative evidence of inequalities in access to healthcare among ASR who are subject to the three different access models. The aim of our research was therefore to analyze if different access models (HV, EHC, regular access) are associated with inequalities in access to healthcare understood as realized access or forgone care.

## Methods

### Design, sampling, and population

We used data from three population-based, cross-sectional surveys among newly arrived ASR (N=863) living in accommodation or reception centres in the states of Baden-Württemberg (BW) and Berlin (BE). In BE, the EHC was introduced in 2016; whereas in BW, all municipalities use HVs [11]. Sampling, recruitment, and survey instruments were nearly identical in both states [26, 27]. Around 3% of all 2,017 accommodation centres across the two states (n=81) were selected using random sampling and all adult residents of these centres (census approach) were invited to participate in the survey. In addition, six reception centres from BW were purposively selected for inclusion, with 25% of residents selected by random sampling and invited to participate. The overall response rate was 30.5% (see additional file 1). Questionnaires were developed from standardised, international survey instruments. They covered health status, access to and utilization of health services and socio-demographic aspects. Participants filled out a paper questionnaire in one of nine languages. Data collection for the majority of respondents (96% of the sample) took place between January 2018 and November 2018, while less than 4% of participants (32 persons) were recruited in December 2019. The study design, sampling procedure and data collection process have been described in more detail elsewhere [26, 27].

### Outcome measures

A wide range of utilization (or process) indicators have been suggested to measure realized access [6, 7]. While utilization indicators are important to detect barriers and assess equity in access to health services, utilization is not always an aim in itself [8]. Therefore, in addition to utilization indicators, outcome indicators related to the health consequences of service utilization (vs. forgone or delayed care) are also commonly included in the measurement of access. Subjective unmet needs and avoidable hospitalizations are two important outcome indicators that are internationally used to this end [10, 28]. They have been adapted to the German context [29, 30] and to refugee populations in Germany in particular [31–33]. Subjective unmet need describes a situation in which healthcare was not sought despite subjectively felt need [28]. Avoidable hospitalization can be defined as hospital admissions for conditions for which hospitalization can be prevented by providing timely and adequate treatment in the outpatient setting. These conditions are defined as ambulatory care-sensitive conditions (ACSC) [10].

To analyse differences in access to healthcare, we included three utilization and three outcome indicators. As utilization indicators we included self-reported utilization of general practitioner (GP) and specialist services in the last four weeks (y/n) and of emergency departments in the last 12 months (y/n). As outcome indicators we included hospital admissions for ACSC in the last 12 months (y/n) and subjective unmet needs for specialist or GP services in the last 12 months (y/n). ACSC were assessed using two questions: first, participants were asked whether they had one of the conditions identified as ACSC by Sundmacher et al. [29] in the last 12 months (see additional file 2). Second, they were asked whether they had been hospitalized for any of the said conditions. To assess unmet needs, participants were asked directly if they had refrained from seeking healthcare despite the subjectively felt need to see a doctor.

### Exposures and Co-variables

The access model used – HV, EHC or regular access – was set as the exposure. It was directly assessed for the state of BW. For BE, all persons with a duration of stay of more than 15 months or with a secured residence status (refugee status or subsidiary status) were coded as having regular access, while all others were considered using an EHC. It is important to note that this “waiting period” was extended from 15 to 18 months in August 2019. That is, at the time of data collection, restricted health entitlements applied during asylum seekers’ first 15 months in Germany. For this reason, we used a duration of stay of 15 months as a cut-off date in our data analysis.

A conceptual approach to identifying major determinants of health service utilization and to differentiating between determinants of inequalities in realized access has been developed by Andersen and colleagues. They distinguish between predisposing characteristics (e.g., age, sex, socioeconomic status) and enabling resources which are related to the health system (e.g., health policy, financing, organization, resources, and availability of services). Given the importance of the actual health status and related healthcare needs as a major determinant of healthcare utilization, it is essential to include the actual health status in our analyses to approximate and adequately adjust for underlying healthcare needs in the study population [5, 6, 34]. We therefore included major predisposing characteristics (age, sex, region of origin, duration of stay in Germany, accommodation type and education) and important need- and health-related information (subjective health, chronic illness, and having a regular GP) as covariates.

In order to consider differences in access related to geographical factors, like urban-rural characteristics, we performed a sensitivity analysis by adjusting all full models additionally for urbanity. Districts with a population density below 150 inhabitants per km^2^ were categorised as rural, those with higher density as urban, following the definition of the Federal Institute for Research on Building, Urban Affairs and Spatial Development (BBSR )[35]. As BE is considered as urban in its entirety, the approach primarily controlled for variations in access between rural areas in the state of BW and urban areas in BW and BE.

### Statistical analysis

We used logistic regression to calculate odds ratios (OR) for all outcomes, adjusting for socio-demographic characteristics (age, sex, region of origin, duration of stay in Germany, accommodation type and education) and health-related covariates (subjective health, chronic illness and having a regular GP) which we identified as potential confounders. Having regular access – as compared to access via EHC or HV – was chosen as a reference category. Based on the literature, this was considered the best possible access option. In addition, the HV model was also used as reference category repeating the same regression analyses for all outcomes. This allowed for a direct comparison of differences in access between the HV and EHC model.

All analyses were weighted (using design and calibration weights), treating reception centres in BW, accommodation centres in BW, and accommodation centres in BE each as separate clusters (see additional file 3). Calibration was conducted using data from the statistical offices in BW and BE for age, sex and region of origin [36, 37]. Missing values did not show systematic patterns related to the outcome and were thus assumed to be missing at random. For outcome and exposure indicators, missing values were imputed using single imputation according to the R-package *mice* [38] (see additional file 4). To understand the sensitivity of our results to weighting, the design effect (DEFF) was calculated. Low DEFF indicate small weighting effects. The overall model fit comparing the differences between observed and expected values for the Null-model and the full model was assessed using an adapted F-test for weighted survey designs. Larger F-values with non-significant p-values (>0.05) indicate better model fit [39].

## Results

### Descriptive results

The sample includes responses from 863 individuals of which 560 were living in the state of BW and 303 in the state of BE. Of the 560 participants in BW, 250 (44.6%) were using the HV model and 240 (42.7%) reported regular access. For 70 individuals (12.5%), information on the access model was missing. In Berlin, 49 (16.2%) were using the EHC model and 227 (74.9%) were having regular access while information on the access model was missing for 27 participants (8.9%).

There were no significant differences in age, sex, educational score or health status (subjective health or chronic illnesses) between persons subject to different access models. Given the requirements for regular access (either duration of stay of more than 15 months at the time of data collection or refugee status), duration of stay and residence status are highly associated with the access model. The region of origin is also significantly associated with the access model (c. Tab. 2 for details).

**Table 2:** Socio-demographic and health-related information of the sample according to access model

	HV (BW)		EHC (BE)		Regular access in BW (after HV use)		Regular access in BE (after EHC use)		Missing		Total	
	N	*%*	N	*%*	N	*%*	N	*%*	N	*%*	N	*%*
Total	**250**	*100*	**49**	*100*	**240**	*100*	**227**	*100*	**97**	*100*	**863**	*100*
**Age at interview**												
18-25	**80**	*32*	**9**	*18.4*	**72**	*30*	**47**	*20.7*	**15**	*15.5*	**223**	*25.8*
26-30	**42**	*16.8*	**7**	*14.3*	**35**	*14.6*	**35**	*15.4*	**12**	*12.4*	**131**	*15.2*
31-35	**41**	*16.4*	**8**	*16.3*	**39**	*16.3*	**30**	*13.2*	**8**	*8.2*	**126**	*14.6*
36-40	**31**	*12.4*	**7**	*14.3*	**34**	*14.2*	**28**	*12.3*	**4**	*4.1*	**104**	*12.1*
41+	**32**	*12.8*	**8**	*16.3*	**49**	*20.4*	**49**	*21.6*	**5**	*5.2*	**143**	*16.6*
Missing	**24**	*9.6*	**10**	*20.4*	**11**	*4.6*	**38**	*16.7*	**53**	*54.6*	**136**	*15.8*
**Sex**												
Male	**168**	*67.2*	**23**	*46.9*	**155**	*64.6*	**138**	*60.8*	**33**	*34*	**517**	*59.9*
Female	**69**	*27.6*	**17**	*34.7*	**76**	*31.7*	**70**	*30.8*	**17**	*17.5*	**249**	*28.9*
Missing	**13**	*5.2*	**9**	*18.4*	**9**	*3.8*	**19**	*8.4*	**47**	*48.5*	**97**	*11.2*
Educational score*												
Low	**57**	*22.8*	**9**	*18.4*	**64**	*26.7*	**66**	*29.1*	**14**	*14.4*	**210**	*24.3*
Medium	**80**	*32*	**16**	*32.7*	**84**	*35*	**69**	*30.4*	**13**	*13.4*	**262**	*30.4*
High	**52**	*20.8*	**11**	*22.4*	**34**	*14.2*	**46**	*20.3*	**8**	*8.2*	**151**	*17.5*
Missing	**61**	*24.4*	**13**	*26.5*	**58**	*24.2*	**46**	*20.3*	**62**	*63.9*	**240**	*27.8*
Region of origin***												
Eastern Europe	**6**	*2.4*	**0**	*0*	**5**	*2.1*	**9**	*4*	**1**	*1*	**21**	*2.4*
Southern Europe	**12**	*4.8*	**1**	*2*	**5**	*2.1*	**5**	*2.2*	**1**	*1*	**24**	*2.8*
Western Asia	**59**	*23.6*	**21**	*42.9*	**65**	*27.1*	**93**	*41*	**19**	*19.6*	**257**	*29.8*
Southern Asia	**42**	*16.8*	**7**	*14.3*	**77**	*32.1*	**82**	*36.1*	**15**	*15.5*	**223**	*25.8*
Western Africa	**75**	*30*	**1**	*2*	**33**	*13.8*	**2**	*0.9*	**12**	*12.4*	**123**	*14.3*
Central Africa	**5**	*2*	**0**	*0*	**8**	*3.3*	**1**	*0.4*	**1**	*1*	**15**	*1.7*
Northern Africa	**3**	*1.2*	**0**	*0*	**0**	*0*	**1**	*0.4*	**0**	*0*	**4**	*0.5*
Other	**31**	*12.4*	**14**	*28.6*	**38**	*15.8*	**23**	*10.1*	**5**	*5.2*	**111**	*12.9*
Missing	**17**	*6.8*	**5**	*10.2*	**9**	*3.8*	**11**	*4.8*	**43**	*44.3*	**85**	*9.8*
Residence status***												
Asylum seeker	**173**	*69.2*	**28**	*57.1*	**90**	*37.5*	**64**	*28.2*	**24**	*24.7*	**379**	*43.9*
Asylum granted	**10**	*4*	**7**	*14.3*	**66**	*27.5*	**106**	*46.7*	**6**	*6.2*	**195**	*22.6*
Toleration ('Duldung')	**16**	*6.4*	**4**	*8.2*	**24**	*10*	**14**	*6.2*	**2**	*2.1*	**60**	*7*
Asylum status rejected	**28**	*11.2*	**2**	*4.1*	**22**	*9.2*	**20**	*8.8*	**3**	*3.1*	**75**	*8.7*
Missing	**23**	*9.2*	**8**	*16.3*	**38**	*15.8*	**23**	*10.1*	**62**	*63.9*	**154**	*17.8*
Time since arrival (months)***												
0-6	**115**	*46*	**16**	*32.7*	**24**	*10*	**5**	*2.2*	**8**	*8.2*	**168**	*19.5*
6-12	**41**	*16.4*	**14**	*28.6*	**13**	*5.4*	**3**	*1.3*	**3**	*3.1*	**74**	*8.6*
13-15	**45**	*18*	**7**	*14.3*	**50**	*20.8*	**8**	*3.5*	**4**	*4.1*	**114**	*13.2*
16-24	**16**	*6.4*	**0**	*0*	**105**	*43.8*	**61**	*26.9*	**12**	*12.4*	**194**	*22.5*
24+	**2**	*0.8*	**3**	*6.1*	**25**	*10.4*	**90**	*39.6*	**2**	*2.1*	**122**	*14.1*
Missing	**31**	*12.4*	**9**	*18.4*	**23**	*9.6*	**60**	*26.4*	**68**	*70.1*	**191**	*22.1*
**General health**												
Very good	**33**	*13.2*	**10**	*20.4*	**42**	*17.5*	**46**	*20.3*	**13**	*13.4*	**144**	*16.7*
Good	**83**	*33.2*	**11**	*22.4*	**71**	*29.6*	**65**	*28.6*	**25**	*25.8*	**255**	*29.5*
Fair	**73**	*29.2*	**21**	*42.9*	**72**	*30*	**60**	*26.4*	**18**	*18.6*	**244**	*28.3*
Bad	**30**	*12*	**5**	*10.2*	**21**	*8.8*	**30**	*13.2*	**7**	*7.2*	**93**	*10.8*
Very bad	**19**	*7.6*	**1**	*2*	**14**	*5.8*	**7**	*3.1*	**9**	*9.3*	**50**	*5.8*
Missing	**12**	*4.8*	**1**	*2*	**20**	*8.3*	**19**	*8.4*	**25**	*25.8*	**77**	*8.9*
**Longstanding illness**												
No	**136**	*54.4*	**23**	*46.9*	**127**	*52.9*	**127**	*55.9*	**45**	*46.4*	**458**	*53.1*
Yes	**99**	*39.6*	**23**	*46.9*	**90**	*37.5*	**78**	*34.4*	**23**	*23.7*	**313**	*36.3*
Missing	**15**	*6*	**3**	*6.1*	**23**	*9.6*	**22**	*9.7*	**29**	*29.9*	**92**	*10.7*

*** p<0.001, ** p<0.01, * p<0.05

Of all participants, 29% indicated having used specialist services and 43.3% reported having seen a GP in the last four weeks. There was a considerable difference between the states for specialist utilization with only 24.1% reporting a visit to a specialist in BW compared to 38% in BE. We found no substantial differences in emergency department use, subjective unmet needs of specialist and GP services, and avoidable hospitalization. In total, 27.9% reported at least one visit to the emergency department, 26% and 26.7% reported unmet needs for specialist and GP services respectively, and 21.3% reported at least one avoidable hospitalization in the last 12 months. The share of missing information was rather high for specialist utilization (24.1%), but also for GP utilization (18.1%) and unmet needs (19.7% and 18.7%), while it was only 10.7% for emergency department use and 7.1% for avoidable hospitalization (c. Tab. 3).

**Table 3:** Utilization and outcome measures according to state

	Baden-Württemberg		Berlin		Total	
	N	*%*	N	*%*	N	*%*
Specialist utilization last 4 weeks***						
No	**284**	*50.7*	**121**	*39.9*	**405**	*46.9*
Yes	**135**	*24.1*	**115**	*38*	**250**	*29*
Missing	**141**	*25.2*	**67**	*22.1*	**208**	*24.1*
**GP utilization last 4 weeks**						
No	**226**	*40.4*	**107**	*35.3*	**333**	*38.6*
Yes	**236**	*42.1*	**138**	*45.5*	**374**	*43.3*
Missing	**98**	*17.5*	**58**	*19.1*	**156**	*18.1*
**Specialist unmet need last 12 months**						
No	**310**	*55.4*	**159**	*52.5*	**469**	*54.3*
Yes	**139**	*24.8*	**85**	*28.1*	**224**	*26*
Missing	**111**	*19.8*	**59**	*19.5*	**170**	*19.7*
**GP unmet need last 12 months**						
No	**318**	*56.8*	**154**	*50.8*	**472**	*54.7*
Yes	**144**	*25.7*	**86**	*28.4*	**230**	*26.7*
Missing	**98**	*17.5*	**63**	*20.8*	**161**	*18.7*
**Emergency department visit last 12 months**						
No	**364**	*65*	**166**	*54.8*	**530**	*61.4*
Yes	**149**	*26.6*	**92**	*30.4*	**241**	*27.9*
Missing	**47**	*8.4*	**45**	*14.9*	**92**	*10.7*
**Avoidable hospitalization last 12 months**						
No	**393**	*70.2*	**225**	*74.3*	**618**	*71.6*
Yes	**128**	*22.9*	**56**	*18.5*	**184**	*21.3*
Missing	**39**	*7*	**22**	*7.3*	**61**	*7.1*
**Total**	**560**	*100*	**303**	*100*	**863**	*100*

*** p<0.001, ** p<0.01, * p<0.05

### Inequalities in access comparing HV and EHC with regular access

ASR under the HV model were less likely to use specialist (OR=0.46 [0.31-0.70]) or GP (OR=0.57 [0.34-0.95]) services compared to ASR with regular access while adjusting for age and sex. This difference is significant based on the 95%-CI. For the other outcomes, no significant differences could be observed between both groups.

**Fig. 1:**
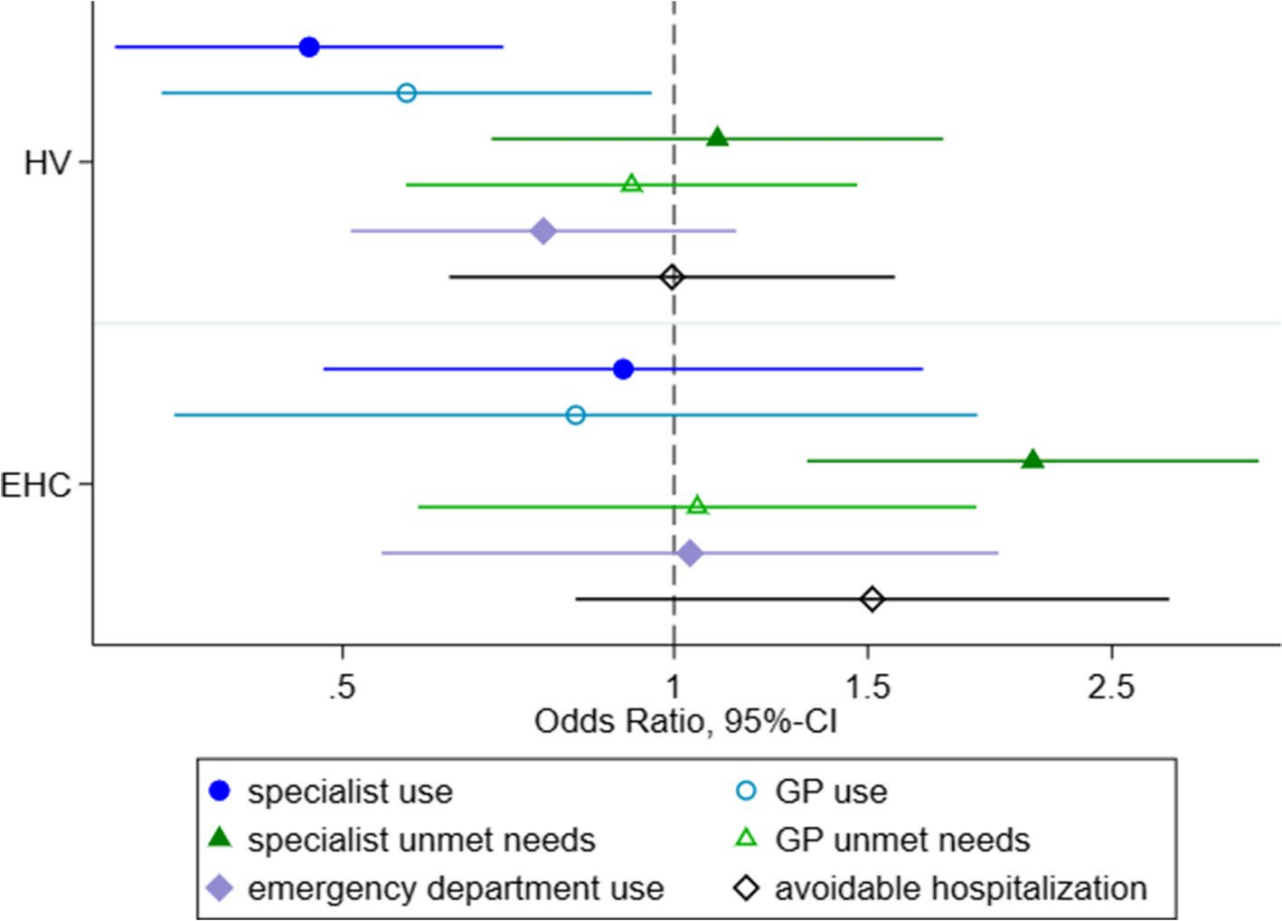
Odds-Ratios (and 95%-CIs) of access to healthcare comparing between access models, adjusted for age and sex (ref=regular access) Legend: HV=Healthcare voucher; EHC=electronic health card; x-axis with 95% confidence intervals on a log-scale

ASR under the EHC model were more likely to report unmet needs for specialist services (OR=2.11 [1.32-3.40]) compared to ASR with regular access while adjusting for age and sex. No significant difference was found for GP and specialist utilization, GP unmet needs, avoidable hospitalization, and emergency department visits between the two groups (c. Fig. 1).

The final models were adjusted for health status, duration of stay, region of origin, educational score, having a regular GP and accommodation type. Their results were similar to those of the simple models. ASR under the HV model showed lower odds of specialist utilization (OR=0.41 [0.24-0.66]) compared to ASR with regular access. For all other indicators, there was no difference between HV users and ASR with regular access.

**Fig. 2:**
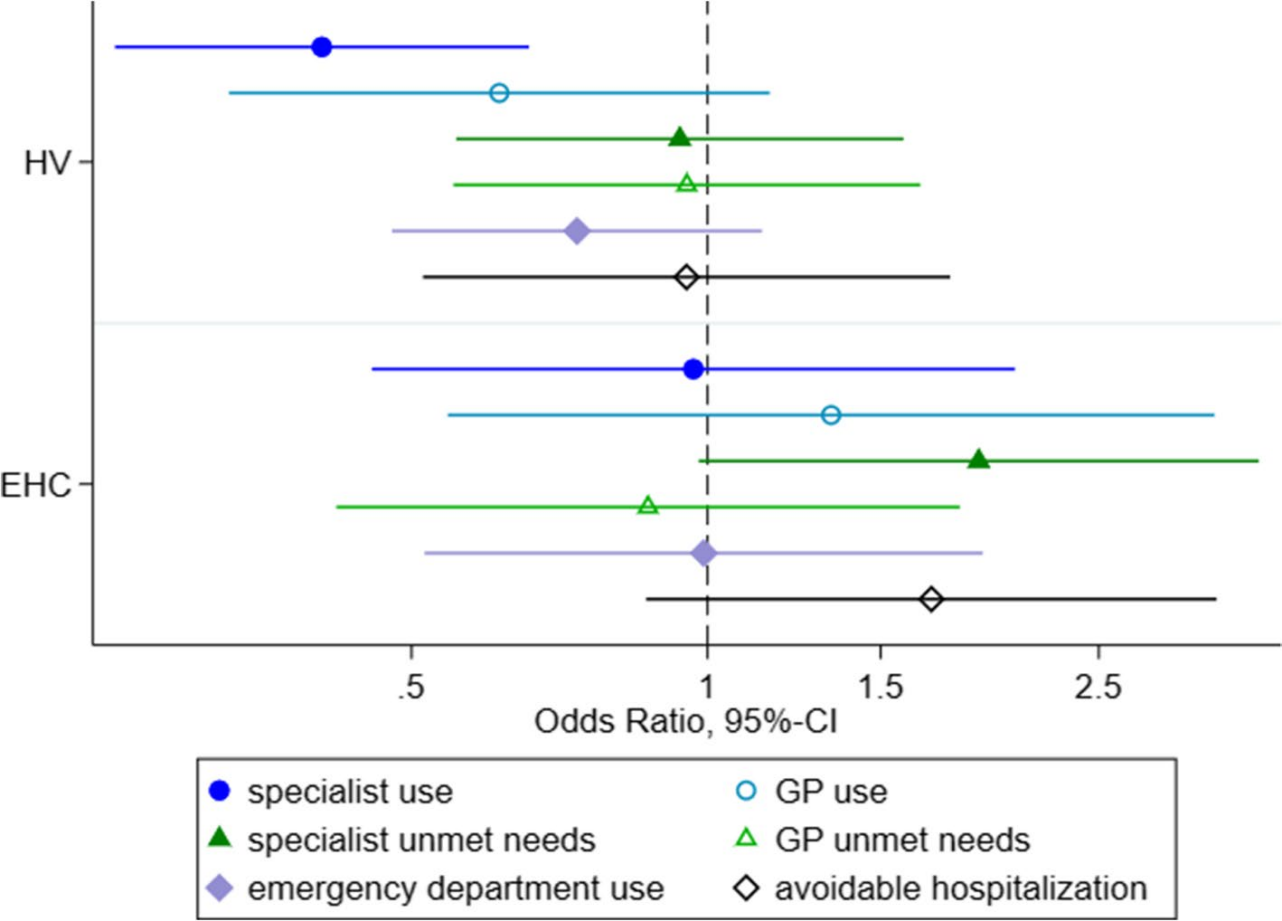
Fully adjusted Odds-Ratios (and 95%-CIs) of access to healthcare comparing between access models (ref=regular access) Legend: HV=Healthcare voucher; EHC=electronic health card; x-axis with 95% confidence intervals on a log-scale

ASR under the EHC model did not show any statistically significant differences (c. Fig. 2).

### Inequalities in access comparing the HV and the EHC model

**Fig. 3:**
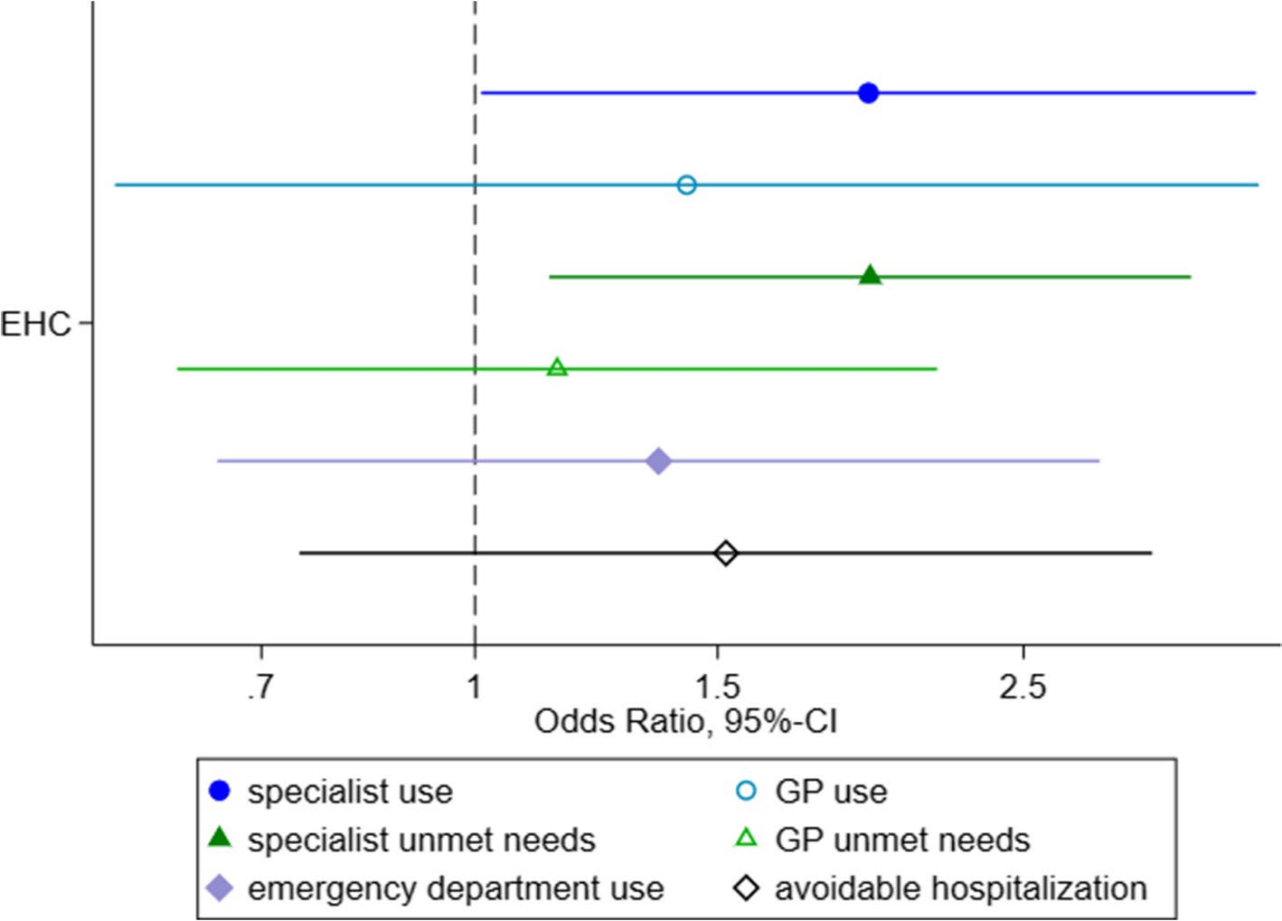
Odds-Ratios (and 95%-CIs) of access to healthcare comparing between access models used in the first 15 months, adjusted for age and sex (ref=HV) Legend: HV=Healthcare voucher; EHC=electronic health card; x-axis with 95% confidence intervals on a log-scale

The comparison of access between the two models that apply during the 15 months waiting period showed higher odds for specialist utilization (OR=1.93 [1.01-3.69]) and specialist unmet needs (OR=1.94 [1.13-3.31]) among ASR under the EHC model compared to ASR under the HV model, adjusting for age and sex. For the remaining four outcomes, odds among ASR with a EHC were also higher compared to odds among ASR with HVs. However, given wide confidence intervals that include the value one, they do not indicate significant differences (c. Fig. 3).

**Fig. 4:**
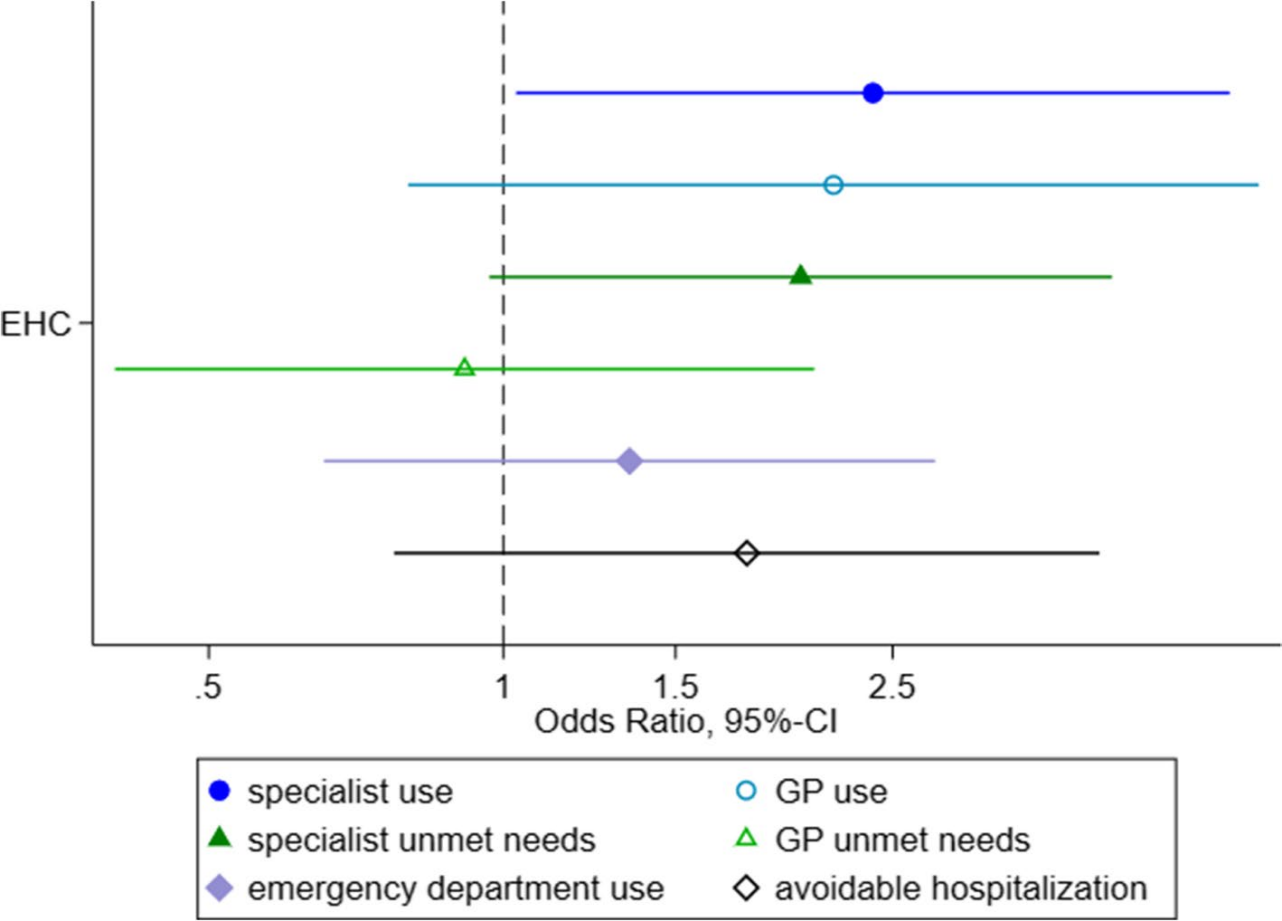
Fully adjusted Odds Ratios (and 95%-CIs) of access to healthcare comparing between access models used in the first 15 months (ref=HV) Legend: HV=Healthcare voucher; EHC=electronic health card; x-axis with 95% confidence intervals on a log-scale

After adjustment for health status and other potential confounders the odds for specialist utilization in the last four weeks were still significantly higher under the EHC model (OR=2.39 [1.03-5.52]) as compared to the HV model. Odds of GP utilization (OR= 2.18 [0.80-5.92]), specialist unmet needs (OR=2.01 [0.97-4.19]), emergency department use (OR= 1.35 [0.66-2.76]) and avoidable hospitalization (OR=1.77 [0.77-4.07]) showed higher point-estimates under the EHC model compared to the HV model. However, 95%-confidence intervals suggest that all differences were not statistically significant (c. Fig. 4).

Overall model-fit was acceptable with non-significant F-tests for nearly all final models (range of *p*-values between 0.420 and 0.849; exception: *p *< 0.001 for avoidable hospitalization). Analysis of the design effects for the final regression models (range of DEFF between 0.993 and 2.520) showed a moderate influence of weighting on the results, which stresses the importance of weighting for valid and generalizable results. However, the comparison of ORs between weighted and non-weighted results did not reveal major differences for any of the outcomes (see Additional files 5, 6, 7, 8, 9). The results of the sensitivity analysis (controlling for urbanity) did neither lead to any considerable changes of the results in terms of magnitude or direction of estimates or statistical significance (Additional Files 10-11), nor did it improve the overall model fit (data not shown).

## Discussion

Our study is the first comparison of realized access to healthcare between the three different access models for ASR in Germany. It thus adds important empirical knowledge to the current literature on access to healthcare among ASR. Our results show significant differences for specialist service utilization between the access models. ASR under the HV model reported lower needs-adjusted utilization of specialist services compared to persons using the EHC and to persons with regular access.

The lower utilization of specialist services might be related to access barriers that are inherent to the HV model (such as the need for prior approval by the local welfare office for specialist utilization, or the limited validity of HVs to three months). For all other outcomes – GP utilization, unmet needs, emergency department use and avoidable hospitalization – differences between the groups were neither consistent nor significant in the fully adjusted models. Tendencies towards differences in GP utilization, specialist unmet needs and avoidable hospitalizations should be further explored.

Using data from three population based, multi-lingual surveys with tested items contributed to the validity of the results. It also enabled us to control for a wide range of socio-demographic health-related, and geographical confounders captured in the survey. The underlying survey data was adequate for our study as we used random sampling techniques, adaptive recruitment and surveying strategies to draw a comprehensive and reliable picture of health and healthcare access among ASR [26]. We obtained a response rate that was comparable to rates obtained in nation-wide surveys of the general population (e.g. 35% in the German Population Survey of the Social Sciences (ALLBUS), 42% in the DEGS survey of the Robert Koch-Institute) [40, 41]. Furthermore, we performed state-of-the-art imputation of missing data to avoid bias through inappropriate use of complete case analyses (i.e. excluding participants with missing observations on given outcomes or co-variables).

Besides these strengths, there are important methodological implications of our research. First, there was a significant association between region of origin and access model. This is mainly explained by the fact that the country of origin influences the distribution of newcomers among states in Germany as well as their chances of obtaining permanent legal status. At the same time, it highlights the importance of controlling for region or country of origin when making comparisons between the German states. Second, to the best of our knowledge, this was the first time that avoidable hospitalizations were assessed in a survey design (and not through routine or claims data using ICD-codes). The approach turned out to be feasible and the comparatively low share of missing responses (only 7.1%) showed a high acceptance among respondents. Future studies should further evaluate the item’s validity and potential for use in future health surveys. This is especially important for research on populations that tend to remain left out of routine data collection.

Third, while the questions referring to specialist and GP service utilization referred to the last four weeks, questions related to all other outcomes referred to the last 12 months. This may have led to a recall bias as people are requested to report their health seeking behaviour for long periods of time. In addition, respondents potentially changed from one access model to another (from HV or EHC to regular access), resulting in misclassification bias. Thus, estimates for unmet needs, emergency department use and avoidable hospitalization are less robust than for specialist and GP utilization. The observed minor differences for these outcomes (e.g., for specialist unmet needs) are therefore not further interpreted. Finally, our data is from two different states with potentially unmeasured differences in availability of interpreters and organization of healthcare services. These unmeasured differences might constitute confounders especially for the comparison between HV and EHC, as all included HV users lived in BW while all EHC users lived in BE.

Our results are in line with qualitative studies that hypothesised lower utilization of outpatient services among HV users due to bureaucratic barriers [19, 20]. A quantitative analysis of claims data [23] and a regional survey [22] in the state of North Rhine Westphalia, too, have identified access barriers related to specialist utilization, which ultimately led to inequalities in healthcare utilization. There is thus reason to suspect that persons who are subject to the HV model have lower access to specialist services compared to EHC users and people with regular access, while having equivalent needs. According to the literature on health inequalities this would constitute a violation of the principle of horizontal equity (equal access for equal needs) [42]. We did not find inequalities related to unmet needs, emergency department use and avoidable hospitalization. Other studies reported significant differences for these outcomes; for example, higher rates of avoidable hospitalizations among EHC users as compared to persons with regular access [32], and higher emergency department use under the HV model as compared to the EHC model [23]. We could not back up these findings with our analyses, which may be due to the abovementioned methodological limitations.

The results of our study have important implications for the controversial debate on the choice of access model for ASR during their first 18 months in Germany. The identified inequalities in access to specialist and GP services provide further evidence for the advantages of the EHC model compared to the HV model. The EHC model facilitates need-based healthcare utilization by providing access similar to the regular access model. Those local governments that, nonetheless, adhere to the HV model often justify their policy decision with cost arguments; that is, with the assumption that the EHC model would lead to excessive utilization of healthcare and thereby increase health expenses [43]. Given that recent studies refute such cost arguments [24, 44], little evidence-based arguments are left to justify upholding the HV model. In that light, policymakers who have so far opted for the HV model may want to reconsider introducing the EHC (or disclose the remaining reasons for not doing so). The relevance of such policy change has increased lately, as in August 2019 the waiting period during which the respective access models (HV and EHC) apply has been prolonged from 15 to 18 months [11].

While this study looked at the direct effects of the different models on access, we were unable to analyse long-term effects of lower healthcare utilization among HV users on their health status. Longitudinal studies will be needed to study the health consequences of the different access models. Such studies could revisit analyses of avoidable hospitalizations, emergency department use and unmet needs, as methodological limitations impeded a thorough analyses of these aspects in our study.

## Conclusion

ASR who are subject to the HV model are disadvantaged in their access to healthcare. With equal need, they use specialist services less often than ASR with an EHC and those with regular access. The identified inequalities constitute inequities in access to healthcare that could be reduced by policy change from HV to the EHC model (or by granting regular access upon arrival). The EHC model ensures access to GP and specialist services comparable to regular access as there are no significant differences in outpatient care utilization between ASR with EHC and ASR with regular access. Interpretation of the results for unmet needs, emergency department use and avoidable hospitalization is limited due to methodological constrains. Still, the respective patterns of difference that were observed deserve further exploration in future studies.

## Supplementary Information

**Additional file 1.**

**Additional file 2.**

**Additional file 3.**

**Additional file 4.**

**Additional file 5.**

**Additional file 6.**

**Additional file 7.**

**Additional file 8.**

**Additional file 9.**

**Additional file 10.**

**Additional file 11.**

## Data Availability

The datasets used during the current study are available from https://respond-study.org/en/resources/ on reasonable request to the corresponding author.

## Abbreviations

ACSC: Ambulatory care sensitive conditions
ASR: Asylum seekers and refugees
AsylbLG: Asylbewerberleistungsgesetz/Asylum Seeker’s Benefits Act
BW: Baden-Wuerttemberg
BE: Berlin
DEFF: Design effect
EHC: Electronic health card
GP: General practitioner
HV: Healthcare voucher
SWO: Social welfare office
