# Supplementary figures and images for: Inequalities in access to healthcare by local policy model among newly arrived refugees: evidence from population-based studies in two German states
Source: Int J Equity Health. 2022 Jan 24;21:11. doi: 10.1186/s12939-021-01607-y (PMC8785512; doi:10.1186/s12939-021-01607-y)

## Additional file 1: Participants' flow diagram and response rate

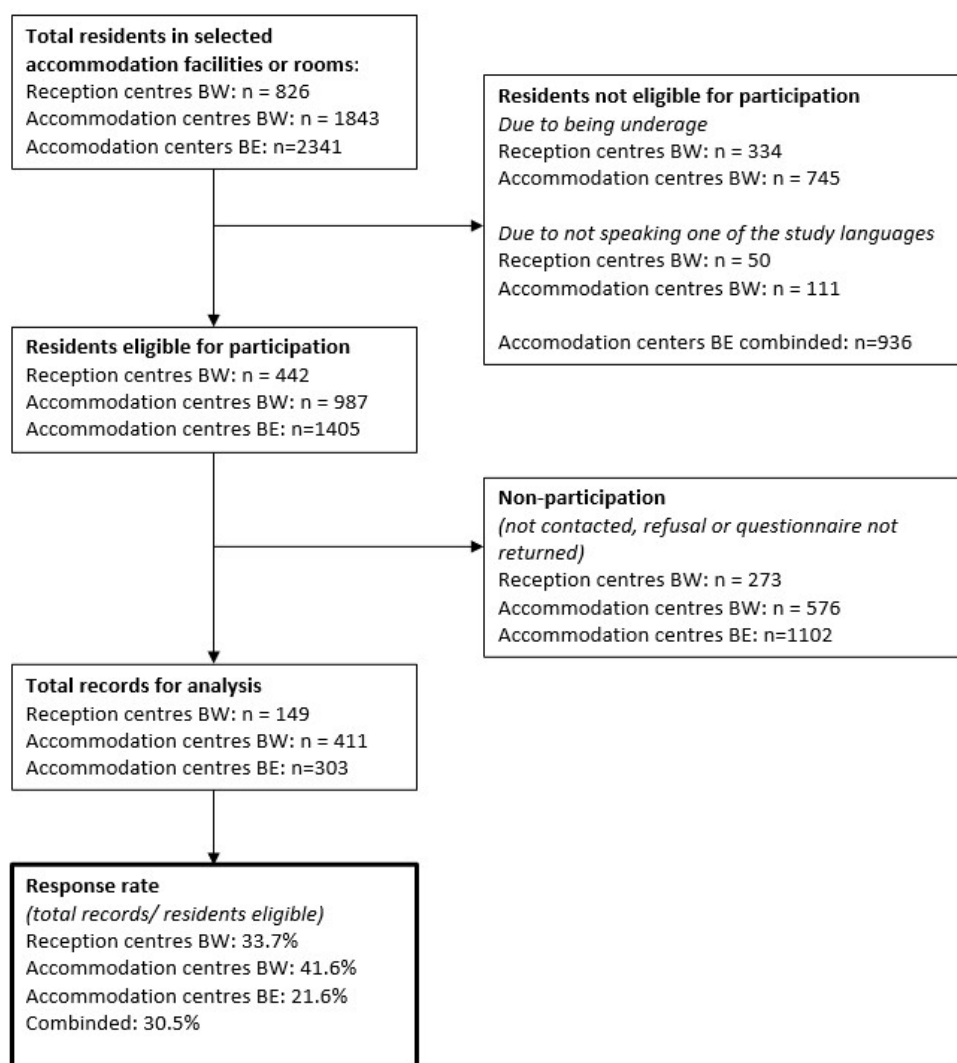

Supplement: Supplementary file 1 — Additional file 1. [file 12939_2021_1607_MOESM1_ESM.pdf]
