# Supplementary material for: Inequalities in access to healthcare by local policy model among newly arrived refugees: evidence from population-based studies in two German states
Source: Int J Equity Health. 2022 Jan 24;21:11. doi: 10.1186/s12939-021-01607-y (PMC8785512; doi:10.1186/s12939-021-01607-y)
Supplement: Supplementary file 2 — Additional file 2. [file 12939_2021_1607_MOESM2_ESM.pdf]

Additional file 2: List of ambulatory care-sensitive condition

Stroke
Angina
Heart failure
High blood pressure
Bronchitis
Mental or behavioural disorders due to substance abuse
Depression
Back pain
Diarrhoea
Flu
Ear, nose, and throat infections
Diabetes
Epilepsy
Sleeping problems
Cavities
