# Supplementary material for: Inequalities in access to healthcare by local policy model among newly arrived refugees: evidence from population-based studies in two German states
Source: Int J Equity Health. 2022 Jan 24;21:11. doi: 10.1186/s12939-021-01607-y (PMC8785512; doi:10.1186/s12939-021-01607-y)
Supplement: Supplementary file 3 — Additional file 3. [file 12939_2021_1607_MOESM3_ESM.pdf]

Additional file 3: Overview of clusters, stratification, and survey weights

Level	ACs BW	RCs BW	ACs BE	Cluster	Stratification (size)	Finite population correction
Level 1	We randomly selected several ACs from all possible units (prob. of inclusion ca. 3%)	We deterministically chose several RCs based on location and size (prob. of inclusion 100%)	We randomly selected several ACs from all possible units (prob. of inclusion ca. 3%)	AC/RC: facility	*Strata1 (BW)*<br>AC: 33<br>RC: 1<br>*Strata2 (BW+BE)*<br>AC-BW: 19<br>RC-BW: 1<br>AC-BE 3	Total no. of AC:<br>BW n=1945<br>BE n=72
Level 2	We attempted to recruit all **individuals** within each facility (prob. of inclusion 100%)	We randomly chose a number of **rooms** in each facility (prob. of inclusion ca. 25%)	We attempted to recruit all **individuals** within each facility (prob. of inclusion 100%)	AC: individuals<br>RC: rooms		Total no. of rooms/individuals in chosen facility (n=1123)

AC=accommodation centre; RC=reception centre, BW=Baden-Wuerttemberg; BE=Berlin
