# Supplementary material for: Inequalities in access to healthcare by local policy model among newly arrived refugees: evidence from population-based studies in two German states
Source: Int J Equity Health. 2022 Jan 24;21:11. doi: 10.1186/s12939-021-01607-y (PMC8785512; doi:10.1186/s12939-021-01607-y)
Supplement: Supplementary file 4 — Additional file 4. [file 12939_2021_1607_MOESM4_ESM.pdf]

Additional file 4: Overview of imputation models

Model variables for imputation	Imputation method
Age	Predictive mean matching
sex	Logistic regression model
Nationality (region of origin)	Polytomous regression model
Educational score	Predictive mean matching
Residence status	Polytomous regression model
Time since arrival (months)	Predictive mean matching
Access model	Polytomous regression model
GP unmet needs	Logistic regression model
Specialist unmet needs	Logistic regression model
GP 4-week-utilization	Logistic regression model
Specialist 4-week-utilization	Logistic regression model
Emergency department use	Logistic regression model
Avoidable hospitalization	Logistic regression model
Chronical illness	Logistic regression model
General health	Polytomous regression model
Family doctor	Logistic regression model
