# Supplementary material for: Inequalities in access to healthcare by local policy model among newly arrived refugees: evidence from population-based studies in two German states
Source: Int J Equity Health. 2022 Jan 24;21:11. doi: 10.1186/s12939-021-01607-y (PMC8785512; doi:10.1186/s12939-021-01607-y)
Supplement: Supplementary file 6 — Additional file 6. [file 12939_2021_1607_MOESM6_ESM.pdf]

Additional file 6: Detailed results related to Figure 2 – Results of logistic regression model (odds ratios and standard errors)

	Specialist use	*GP use*	*Specialist unmet needs*	*GP unmet needs*	*Emergency dept. use*	*Avoidable hospitalization*
HV (ref. regular access)	0.405***	0.614	0.937	0.953	0.736	0.952
	(0.0972)	(0.192)	(0.243)	(0.258)	(0.158)	(0.291)
EHC (ref. regular access)	0.967	1.336	1.887*	0.870	0.991	1.689
	(0.360)	(0.593)	(0.612)	(0.314)	(0.321)	(0.558)
Age	1.001	0.999	1.004	0.990	0.976**	0.991
	(0.00916)	(0.00992)	(0.0101)	(0.0109)	(0.0117)	(0.0116)
Male (ref. female)	1.386*	1.619**	1.054	1.039	2.238***	1.035
	(0.255)	(0.344)	(0.205)	(0.228)	(0.413)	(0.262)
Time since arrival (months)	0.997	1.004	0.999	1.006	0.995	0.998
	(0.00351)	(0.00401)	(0.00307)	(0.00367)	(0.00332)	(0.00356)
Region: Asia (ref. Europe)	1.000	1.880**	1.246	0.928	0.750	1.026
	(0.307)	(0.540)	(0.441)	(0.286)	(0.184)	(0.290)
Region: Africa (ref. Europe)	1.358	1.571	1.232	0.949	0.910	1.563
	(0.515)	(0.538)	(0.550)	(0.334)	(0.332)	(0.631)
Region: Other (ref. Europe)	1.330	1.991*	1.180	1.015	1.229	1.312
	(0.618)	(0.765)	(0.475)	(0.444)	(0.520)	(0.655)

Rather bad general health (ref. rather good)	2.430***	1.854**	2.323***	2.363***	1.499*	1.752**
	(0.478)	(0.504)	(0.437)	(0.576)	(0.345)	(0.454)
No chronical illness (ref. yes)	1.099	2.516***	2.069***	2.482***	1.789**	3.103***
	(0.223)	(0.681)	(0.458)	(0.600)	(0.436)	(0.784)
Education: low (ref. high)	1.318	0.868	1.009	0.644*	1.133	1.233
	(0.332)	(0.201)	(0.246)	(0.152)	(0.320)	(0.325)
Education: middle (ref. high)	1.412	0.933	0.907	0.821	0.763	0.749
	(0.317)	(0.243)	(0.195)	(0.195)	(0.203)	(0.206)
No family doctor (ref. yes)	1.931***	3.209***	0.643**	0.922	1.886***	1.798***
	(0.451)	(0.663)	(0.120)	(0.186)	(0.414)	(0.337)
Reception centre (ref. accommodation centre)	1.498	1.975**	0.776	0.819	0.870	0.755
	(0.415)	(0.606)	(0.336)	(0.305)	(0.244)	(0.269)
Constant	0.166***	0.100***	0.300	0.436	0.766	0.179**
	(0.105)	(0.0657)	(0.224)	(0.302)	(0.467)	(0.124)
Observations	863	863	863	863	863	863
p-value (F-test)	0.479	0.779	0.420	0.681	0.849	>0.001

*Standard errors in parentheses; *** p<0.01, ** p<0.05, * p<0.1*
