# Supplementary material for: Inequalities in access to healthcare by local policy model among newly arrived refugees: evidence from population-based studies in two German states
Source: Int J Equity Health. 2022 Jan 24;21:11. doi: 10.1186/s12939-021-01607-y (PMC8785512; doi:10.1186/s12939-021-01607-y)
Supplement: Supplementary file 7 — Additional file 7. [file 12939_2021_1607_MOESM7_ESM.pdf]

Additional file 7: Detailed results related to Figure 3 – Results of logistic regression model (odds ratios and standard errors)

	Specialist use	*GP use*	*Specialist unmet needs*	*GP unmet needs*	*Emergency dept. use*	*Avoidable hospitalization*
EHC (ref. HV)	1.930**	1.425	1.935**	1.147	1.359	1.521
	(0.618)	(0.673)	(0.513)	(0.360)	(0.495)	(0.536)
Age	1.015*	1.028***	1.018*	1.012	0.995	1.015
	(0.00852)	(0.0101)	(0.00896)	(0.00948)	(0.00918)	(0.00965)
Male (ref. female)	1.507**	1.719***	1.106	1.106	2.319***	1.145
	(0.270)	(0.334)	(0.207)	(0.218)	(0.419)	(0.254)
Constant	0.217***	0.309***	0.288***	0.315***	0.419**	0.204***
	(0.0697)	(0.104)	(0.0949)	(0.0951)	(0.142)	(0.0598)
Observations	863	863	863	863	863	863
p-value (F-test)	0.990	0.708	0.567	0.945	0.083	0.191

*Standard errors in parentheses; *** p<0.01, ** p<0.05, * p<0.1*
