# Supplementary material for: Inequalities in access to healthcare by local policy model among newly arrived refugees: evidence from population-based studies in two German states
Source: Int J Equity Health. 2022 Jan 24;21:11. doi: 10.1186/s12939-021-01607-y (PMC8785512; doi:10.1186/s12939-021-01607-y)
Supplement: Supplementary file 9 — Additional file 9. [file 12939_2021_1607_MOESM9_ESM.pdf]

Additional file 9: Overview of effect of weighting on main outcomes in final regression models (design effects/DEFF)

	Specialist use	*GP use*	*Specialist unmet needs*	*GP unmet needs*	*Emergency dept. use*	*Avoidable hospitalization*
HV (ref. regular access)	1.485	2.520	1.792	1.911	1.260	1.929
EHC (ref. regular access)	1.216	1.596	1..137	1.172	1.032	0.993
EHC (ref. HV)	1.257	1.746	1.174	1.259	1.038	1.229
