# Supplementary material for: Inequalities in access to healthcare by local policy model among newly arrived refugees: evidence from population-based studies in two German states
Source: Int J Equity Health. 2022 Jan 24;21:11. doi: 10.1186/s12939-021-01607-y (PMC8785512; doi:10.1186/s12939-021-01607-y)
Supplement: Supplementary file 11 — Additional file 11. [file 12939_2021_1607_MOESM11_ESM.pdf]

Additional File 11: Fully adjusted Odds Ratios (and 95%-CIs) of access to healthcare comparing between access models used in the first 15 months (ref=HV), with urban/rural

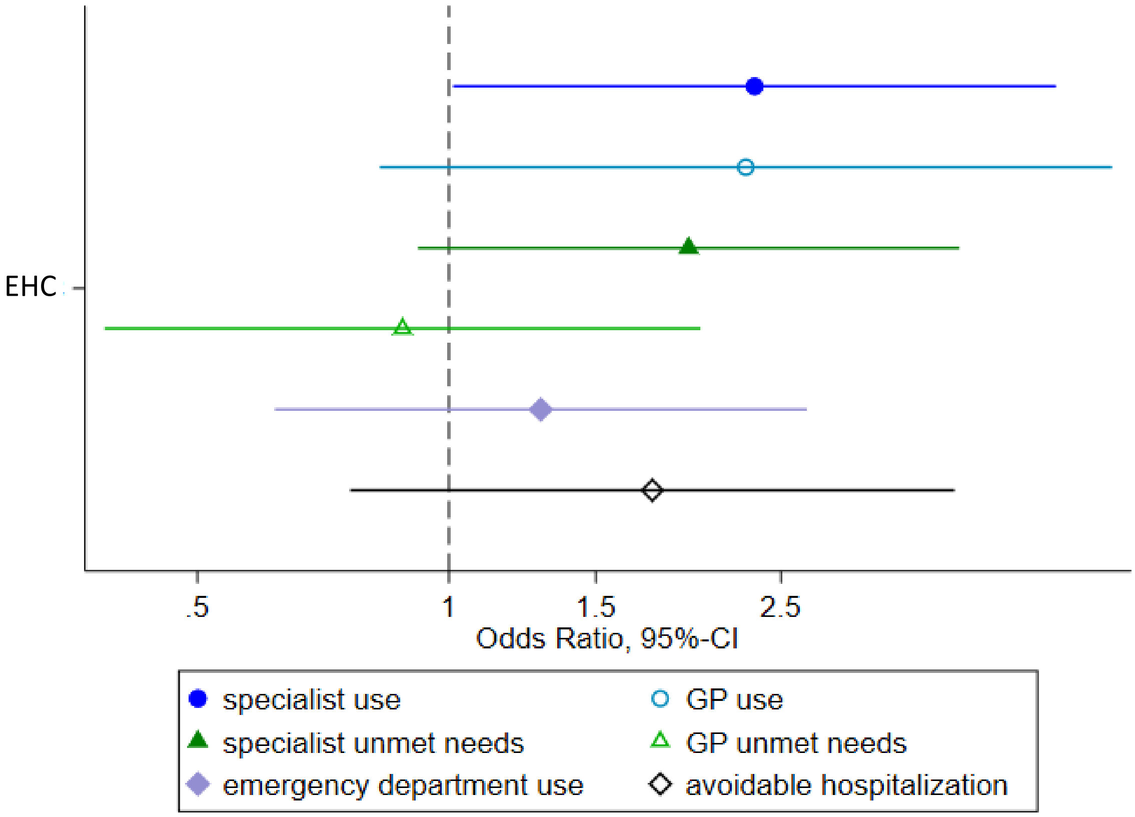

Legend: HV=Health care voucher; EHC=electronic health card; x-axis with 95% confidence intervals on a log-scale
